# Cationic Europium Complexes for Visualizing Fluctuations in Mitochondrial ATP Levels in Living Cells

**DOI:** 10.1002/chem.201801008

**Published:** 2018-06-28

**Authors:** Romain Mailhot, Thomas Traviss‐Pollard, Robert Pal, Stephen J. Butler

**Affiliations:** ^1^ Department of Chemistry Loughborough University Epinal Way Loughborough LE11 3TU UK; ^2^ Department of Chemistry Durham University South Road Durham DH1 3LE UK

**Keywords:** adenosine triphosphate (ATP), anion receptor, lanthanide, live cell imaging, luminescence

## Abstract

The ability to study cellular metabolism and enzymatic processes involving adenosine triphosphate (ATP) is impeded by the lack of imaging probes capable of signalling the concentration and distribution of intracellular ATP rapidly, with high sensitivity. We report here the first example of a luminescent lanthanide complex capable of visualizing changes in the concentration of ATP in the mitochondria of living cells. Four cationic europium(III) complexes [**Eu.1**–**4**]^**+**^ have been synthesized and their binding capabilities towards nucleoside polyphosphate anions examined in aqueous solution at physiological pH. Complexes [**Eu.1**]^+^ and [**Eu.3**]^+^ bearing hydrogen bond donor groups in the pendant arms showed excellent discrimination between ATP, ADP and monophosphate species. Complex [**Eu.3**]^+^ showed relatively strong binding to ATP (log*K*_a_=5.8), providing a rapid, long‐lived luminescent signal that enabled its detection in a highly competitive aqueous medium containing biologically relevant concentrations of Mg^2+^, ADP, GTP, UTP and human serum albumin. This Eu^III^ complex responds linearly to ATP within the physiological concentration range (1–5 mm), and was used to continuously monitor the apyrase‐catalyzed hydrolysis of ATP to ADP in vitro. We demonstrate that [**Eu.3**]^+^ can permeate mammalian (NIH‐3T3) cells efficiently and localize to the mitochondria selectively, permitting real‐time visualization of elevated mitochondrial ATP levels following treatment with a broad spectrum kinase inhibitor, staurosporine, as well as depleted ATP levels upon treatment with potassium cyanide under glucose starvation conditions.

## Introduction

Adenosine triphosphate (ATP) is the most abundant nucleoside polyphosphate (NPP) anion in cells^1^, ^2^ and serves as the chemical energy source for most biological processes, including organelle transport, muscle contraction and maintenance of neuronal membrane potential.^2^, ^3^, ^4^ Despite its importance, there are surprisingly few imaging probes capable of signalling the presence of ATP rapidly, reversibly and selectively under physiological conditions.^5^, ^6^ We have addressed this challenge by creating a luminescent europium(III) complex capable of imaging dynamic changes in the concentration of ATP in the mitochondria of living cells. The majority of ATP is generated by the mitochondria by oxidative phosphorylation. Numerous enzymes utilize ATP as a substrate, including ATPases, kinases, and RNA polymerases; thus ATP plays a key role in signalling and the regulation of enzyme function.^1^, ^2^, ^6^ The release of ATP to the extracellular space has been identified in both damaged and apoptotic cells.^2^ Extracellular ATP also serves as a signalling molecule by binding to purinergic receptors,^3^ thereby mediating an immunological or nervous response. The concentration of ATP varies considerably, from nanomolar extracellular concentrations to millimolar concentrations in the cytosol and certain organelles (e.g. mitochondria).^1^, ^2^ To gain a better understanding of the wide range of dynamic processes involving ATP, non‐invasive imaging probes are required that can signal changes in ATP levels by modulation of luminescence, thereby providing spatial and temporal information rapidly, with high sensitivity.5a, 6a

Current methods for monitoring the concentration of ATP in living cells have limitations.^2^, 5a The enzyme firefly luciferase can be expressed in cells to measure ATP levels indirectly, by catalyzing a chemiluminescent reaction between ATP and luciferin.^7^ However, this method consumes ATP, which may perturb the cellular energy status, preventing accurate quantification of ATP.^8^, ^9^ Genetically encoded fluorescent biosensors (e.g. ATeam,6a PercevalHR)6b have been developed to measure ATP successfully within specific cellular compartments. However, biosensors encoded within cells can require time consuming protein expression and maturation procedures and are intrinsically pH sensitive;6b small changes in intracellular pH (<0.1 units) have been shown to generate bias in the emission response, which can complicate interpretation of the observed signal.^9^

An attractive alternative strategy involves the creation of discrete ATP‐responsive synthetic receptors that exhibit intrinsic cellular uptake and localization behaviour.^1^, 5a The majority of synthetic receptors for ATP comprise a fluorescent organic indicator linked to a positively charged recognition group, such as imidazolium or guanidinium units, or coordinated zinc(II) ions, which form strong electrostatic or metal‐ligand interactions with the triphosphate fragment of ATP.^10^ Only a few ATP‐selective probes have been successfully applied to imaging ATP in living cells.5a–5d Each of these probes emits a short‐lived fluorescence signal, typically at wavelengths between 375 and 540 nm. Consequently, their signal overlaps with background autofluorescence arising from endogenous molecules, which can complicate intensity‐based measurements.^11^ In two cases, the detection range of the receptor is very low (0.1–10 μm),5a,5b and the fluorescence response is saturated at ATP concentrations below those expected in cells (1–5 mm).^12^ Chang and co‐workers developed a probe capable of detecting ATP within the 2–10 mm range, in a viscous medium of glycerol/water (60:40).5c However, this probe is unable to respond to ATP in 100 % aqueous solution at physiological pH, hence its utility in live‐cell imaging is limited and dependent on variations in viscosity within the cell.

Importantly, during in vitro testing, anion affinity and selectivity is rarely assessed in a competitive ionic medium that resembles intracellular fluid.5c This is critical, because it defines the target concentration range and the nature and abundance of potentially interfering species, including other phosphoanions (e.g. ADP, AMP, GTP, HPO_4_^2−^), cations that bind strongly to ATP (e.g. Mg^2+^ and Ca^2+^ ions), and proteins, which can interact with the probe causing either quenching or enhancement of emission. The influence of such a competitive ionic environment on the probe's anion affinity and selectivity should be studied, to optimize its practical utility in live‐cell imaging experiments.^13^

In recent years, stable luminescent europium(III) and terbium(III) complexes have emerged as attractive tools for use in cellular imaging.^14^ Notably, a series of the brightest Eu^III^ complexes have been developed as efficient cellular stains, designed to localize preferentially in specific organelles.^15^, ^16^ Stable Eu^III^ and Tb^III^ complexes offer significant advantages over conventional fluorescent organic dyes, including a large separation between the absorption and emission bands, long emission lifetimes^17^ (within the millisecond range) that enable complete removal of background autofluorescence by using time‐resolved imaging techniques, and well‐defined emission bands that permit ratiometric analysis.^18^ Certain Eu^III^ and Tb^III^ complexes have been shown to bind reversibly to anions in aqueous media, including bicarbonate,^19^ fluoride,^20^ lactate_,_ 21 phosphate^22^ and phosphorylated peptides.^23^ Anion binding can be signalled by changes in the intensity ratio of two Ln^III^ emission bands or by changes in emission lifetime of the complex.^13^, ^24^ However, examples of Ln^III^ complexes that bind to NPP anions such as ATP are rare.^25^, ^26^, ^27^, ^28^ Binding to ATP is usually weak and causes quenching of luminescence due to energy transfer to the nucleotide base^25^, ^27^ or displacement of a coordinated sensitizing ligand from the Ln^III^ ion (decomplexation).^28^, ^29^ Probes that utilise quenching of luminescence are less desirable for use in cellular imaging as other competitive quenching processes may generate the observed decrease in emission intensity.14b

**Figure 1:**
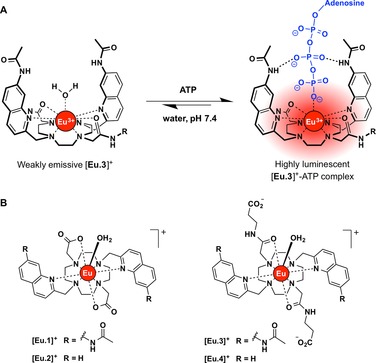
(A) Signalling mechanism and proposed binding mode between **[Eu.3**]^+^ and ATP; (B) Structures of cationic Eu^III^ complexes [**Eu.1**–**4]^+^**.

In this work, we report a series of luminescent cationic Eu^III^ complexes [**Eu.1**–**4**]^+^ that bind reversibly and with differential affinities to NPP anions in aqueous solution at physiological pH (Figure 1). Each Eu^III^ complex is based on a *C*_2_‐symmetric octadentate ligand bearing two coordinating quinoline groups that sensitize Eu^III^ luminescence. Addition of ATP to complexes [**Eu.1**]^+^ and [**Eu.3**]^+^ results in rapid displacement of the coordinated water molecule, giving rise to dramatic enhancements in Eu^III^ luminescence. Complex [**Eu.3**]^+^ is very effective at binding to ATP in water at physiological pH, by means of a strong metal‐ligand interaction, possibly strengthened by hydrogen bonds to the quinoline amide arms. [**Eu.3**]^+^ can discriminate effectively between ATP and ADP in the presence of high concentrations of Mg^2+^ ions, permitting real‐time analysis of the enzymatic hydrolysis of ATP to ADP in vitro. We demonstrate that [**Eu.3**]^+^ can signal changes in the ratio of ATP/ADP in a simulated intracellular fluid containing biorelevant concentrations of several NPP anions including GTP and UTP, as well as Mg^2+^ ions and protein. [**Eu.3**]^+^ was shown to permeate NIH‐3T3 cells efficiently and localize selectively to the mitochondria, providing a strong luminescent signal that can report on dynamic changes in mitochondrial ATP levels within living cells. This new class of Eu^III^ complexes offers a number of improvements in performance compared to existing probes for ATP, including a long‐lived luminescence signal that is sensitive to ATP levels within the physiological range (1–5 mm), with minimal interference from biomolecule autofluorescence or changes in cellular pH. In addition, [**Eu.3**]^+^ possesses a distinctive subcellular localization profile that allows ATP levels to be monitored within a specific region of the cell.

## Results and Discussion

### Complex design and synthesis

Each Eu^III^ complex contains two coordinating quinoline groups that act as efficient sensitizers of Eu^III^ emission.^30^ Complexes [**Eu.1**]^+^ and [**Eu.2**]^+^ were synthesised previously20a and possess two negatively charged carboxylate groups that coordinate to the Eu^III^ ion, whereas complexes [**Eu.3**]^+^ and [**Eu.4**]^+^ possess two neutral carbonyl amide donor groups, functionalized with terminal carboxylate moieties to increase water solubility. The carbonyl amide groups in [**Eu.3**]^+^ and [**Eu.4**]^+^ were expected to increase the electropositive nature of the Eu^III^ metal compared to [**Eu.1**]^**+**^ and [**Eu.2**]^+^, thus strengthening the electrostatic interaction between the Eu^III^ ion and negatively charged NPP anions in aqueous solution. Additionally, it was envisaged that the increased local positive charge of [**Eu.3**]^+^ and [**Eu.4**]^+^, together with the amphipathic nature of the surrounding ligands, would facilitate cellular uptake of the Eu^III^ complexes, and potentially promote selective localization to the negatively charged inner membrane of the mitochondria.^16, 5a^

Complexes [**Eu.1**]^+^ and [**Eu.3**]^+^ possess hydrogen‐bond donor (amide) groups at the 7‐position of the coordinated quinoline chromophores. We envisaged that NPP anions such as ATP would coordinate to the Eu^III^ ion via the terminal phosphate group, enabling the adjacent phosphate group to engage in hydrogen bonding interactions with quinoline amide N‐H groups (Figure 1 B), thereby enhancing selectivity over monophosphate species. Indeed, we have shown recently that the cooperative use of metal‐ligand interactions in combination with hydrogen bonding can provide excellent selectivity for nucleoside polyphosphate anions over monophosphate anions (e.g. AMP, cAMP, HPO_4_^2−^, phosphorylated amino acids), where no such hydrogen‐bonding interactions can occur.^31^, ^32^ To investigate the function of the hydrogen bond‐donor groups in the ATP recognition process, we prepared two control complexes, [**Eu.2**]^+^ and [**Eu.4**]^+^, which lack quinoline amide groups.

Details of the synthesis and characterization of complexes [**Eu.3**]^+^ and [**Eu.4**]^**+**^ are provided in the supporting information (Figure S1–S3 for synthetic schemes). Briefly, a cyclen derivative bearing two *trans*‐related secondary amines was reacted with an appropriately functionalized 2‐methylquinoline mesylate ester or 2‐(chloromethyl)quinoline in the presence of K_2_CO_3_, to give the protected macrocyclic ligand. The terminal ethyl ester protecting groups of the two carbonyl amide arms were hydrolyzed using 0.5 m NaOH solution. Subsequent addition of one equivalent of EuCl_3_ in a mixture of water/methanol at pH 7–8 gave the water soluble Eu^III^ complexes [**Eu.3**]^+^ and [**Eu.4**]^+^, after purification by preparative reverse‐phase HPLC.

**Figure 2:**
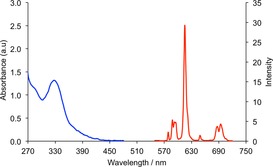
Absorption (*blue*) and emission (*red*) spectra of [**Eu.3**]^+^, recorded in buffered aqueous solution (10 mm HEPES) at pH 7.0. *λ*_exc_=330 nm, 25 °C.

Table 1 shows selected photophysical data for [**Eu.1**–**4**]^+^. The UV/Vis absorption spectra of [**Eu.1**]^+^ and [**Eu.3**]^+^ are similar and comprise of a broad band centred at 332 nm and 330 nm, respectively, tailing out to 370 nm, whereas [**Eu.2**]^+^ and [**Eu.4**]^+^ each feature two narrow bands with maxima at 303 and 318 nm (Figure S4‐S7). The emission spectrum of each complex in aqueous buffer (10 mm HEPES, pH 7.4) was well separated from the absorption spectrum, as illustrated by [**Eu.3**]^+^ in Figure 2. The emission spectra of [**Eu.1**]^+^, [**Eu.2**]^+^ and [**Eu.4**]^+^ showed similarities, displaying at least three components in the Δ*J*=1 (585–605 nm) band, and two distinguishable components within the Δ*J*=2 (605–630 nm) band, consistent with each complex adopting a structure of low symmetry in water (Figure S8). For [**Eu.3**]^+^, a rather different emission spectrum was obtained, characterised by a pronounced Δ*J*=2 band around 605–630 nm and two discernable components within the Δ*J*=4 band around 675–705 nm in the red region of the visible spectrum (Figure 2). Emission quantum yields were in the range 7–23 % and emission lifetimes in H_2_O were measured to be approximately 0.5 ms, and at least 50 % larger in D_2_O. The number of coordinated water molecules, *q*, was determined to be one for each Eu^III^ complex.^33^ These data suggested that complexes [**Eu.1**]^+^ and [**Eu.3**]^+^ were most suited for use in live‐cell imaging experiments, due to their water solubility, long‐lived luminescence and sufficiently long excitation wavelengths of over 350 nm, thereby matching the optics of standard fluorescence microscopes.

**Table 1:** Selected photophysical data for complexes [**Eu.1**–**4**]^+^, measured in 10 mm HEPES, pH 7.0, 25 °C.

Complex	*λ*_max_ [nm]	*ϵ* [m ^−1^ cm^−1^]	*φ*_em_ [%]^[a]^	*τ*_(H2O)_ [ms]	*τ*_(D2O)_ [ms]	*q* ^[b]^
[**Eu.1**]^+^	332	0.00125	7.0	0.49	1.38	1.2
[**Eu.2**]^+^	318	0.00118	23	0.51	1.37	1.1
[**Eu.3**]^+^	330	0.00077	6.5	0.56	1.22	0.8
[**Eu.4**]^+^	318	0.00210	18	0.60	1.29	0.7

[a] Errors in quantum yields and lifetimes are ±20 %; [b] Values of hydration state, *q* (±20 %) were derived using literature methods.^33^

### Anion binding studies at physiological pH

The Eu^III^ emission spectra of [**Eu.1**–**4**]^+^ were recorded in pH 7.0 (10 mm HEPES) in the presence of a range of anions (1 mm) (Figure S9–S12). Addition of 1 mm ATP to [**Eu.1**]^+^ or [**Eu.3**]^+^ resulted in an immediate 10‐fold enhancement in Eu^III^ emission intensity of the hypersensitive Δ*J*=2 band (605–630 nm), whereas adding ADP induced a 14‐fold increase in emission intensity (Figure S9 and S10). AMP and HPO_4_^2−^ induced a much smaller (approximately 2‐fold) increase in emission intensity while other monophosphate anions including cyclic AMP and phosphorylated amino acids (pSer, pThr, pTyr) induced negligible spectral responses, as did chloride, sulphate, lactate, acetate, glutathione, Na^+^, K^+^, Zn^2+^, Mg^2+^ and Ca^2+^ ions. The guanosine phosphate anions, GTP, GDP and GMP induced similar spectral responses to those observed for ATP, ADP and AMP respectively. The only other anions tested that induced a significant spectral response were citrate and bicarbonate, which caused a maximum 4‐fold and 5‐fold increase in the Δ*J*=2 emission band, respectively. Control complexes [**Eu.2**]^+^ and [**Eu.4**]^+^, which lacked quinoline amide groups, showed a much lower level of discrimination between NPP anions: a maximum 3‐fold enhancement in intensity of the Δ*J*=2 emission band was observed in the presence of 1 mm ATP, ADP and AMP (Figure S11 and S12). Analysis of the relative change in total emission intensity (570–720 nm) resulted in a very similar anion selectivity profile for each Eu^III^ complex (Figure S13–S16).

**Figure 3:**
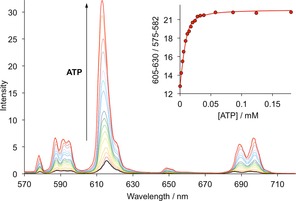
Change in emission spectra of [**Eu.3**]^+^ (5 μm) as a function of added ATP (0–180 μm) in aqueous solution at pH 7.0; (Inset) Binding isotherm and fit to the experimental data, for log*K*_a_=5.8(±0.1). Conditions: 10 mm HEPES buffer, pH 7.0, *λ*_exc_=330 nm, 25 °C.

The emission intensity of [**Eu.3**]^+^ was found to be particularly sensitive to ATP concentration. Figure 3 shows the increase in emission spectra of [**Eu.3**]^+^ in the presence of increasing ATP levels (0–180 μm), revealing a dramatic 17‐fold enhancement in intensity at 614 nm within the Δ*J*=2 band. Notably, the emission spectral shape of [**Eu.3**]^+^ did not change significantly, suggesting that only minor changes in conformation of [**Eu.3**]^+^ occur upon binding to ATP. Emission lifetimes of [**Eu.3**]^+^ measured in H_2_O and D_2_O in the absence and presence of ATP were consistent with a hydration state, *q*, of 0.8(±20 %) and zero, respectively (Table S1), establishing that ATP displaces the coordinated water molecule from the Eu^III^ metal, accompanied by a pronounced increase in luminescence.

Addition of ATP (and ADP) to complex [**Eu.3**]^+^ also caused a considerable increase in intensity of the hypersensitive Δ*J=*4 band (675–705 nm), whereas AMP and HPO_4_^2^ caused much smaller changes (Figure S17 and S18). Moreover, the ATP and ADP adducts of [**Eu.3**]^+^ could be discriminated by the variation in spectral shape of the Δ*J=*4 band; the ATP bound species is characterized by two discernable lines centred at 688 and 697 nm, respectively, whereas the ADP adduct showed at least 4 components within the Δ*J=*4 region. The structurally related complex [**Eu.4**]^+^ showed similar distinctive changes in the shape of the Δ*J=*4 band in the presence of ATP and ADP; however, only minor increases in emission intensity took place (Figures S17 and S18).

Apparent binding constants were determined for complexes [**Eu.1**–**4**]^+^ and a range of NPP anions by following the change in the intensity ratio of the Δ*J*=2/Δ*J*=1 (605–630/580–600 nm) emission bands as a function of anion concentration, followed by a non‐linear, least squares curve‐fitting procedure based on a 1:1 binding model (Table 2 and Figure S19–S49). [**Eu.3**]^+^ showed the strongest binding to ATP and ADP (log*K*_a_=5.8 and 5.7 respectively), approximately 10 times stronger than to AMP and pyrophosphate (PPi), and 100 times stronger than to HPO_4_^2−^ and HCO_3_^−^ (log*K*_a_=2.7 and 3.0 respectively). [**Eu.1**]^+^ showed a similar level of selectivity between ATP, ADP, AMP and PPi, however the affinity for each anion was approximately one order of magnitude lower, and HPO_4_^2−^ was bound weakly (log*K*_a_=2.1). The higher affinity of [**Eu.3**]^**+**^ for ATP compared to [**Eu.1**]^+^ can be attributed to replacement of the negatively charged carboxylate donors in [**Eu.1**]^**+**^ with neutral carbonyl amide donors in [**Eu.3**]^+^, increasing the electropositive nature of the Eu^III^ ion, causing it to interact more strongly with ATP.

**Table 2:** Apparent binding constants (log*K*_a_) determined for [**Eu.1**–**4**]^+^ and different anionic species in aqueous solution (10 mm HEPES, pH 7.0, 25 °C).

Anion	Log *K*_a_^[a]^			
	[**Eu.1**]^+^	[**Eu.2**]^+^	[**Eu.3**]^+^	[**Eu.4**]^+^
ATP	4.4	3.0	5.8	3.8
ADP	4.6	3.3	5.7	3.8
AMP	3.4	3.3	4.8	3.5
PPi	3.5	2.4	4.7	3.4
GTP	4.4	n/d	5.3	n/d
GDP	4.6	n/d	5.2	n/d
GMP	3.4	n/d	3.9	n/d
UTP	3.6	n/d	4.9	n/d
UDP	4.2	n/d	5.4	n/d
UMP	2.7	n/d	4.3	n/d
HPO_4_^2‐^	2.1	n/d	2.7	n/d
HCO_3_^‐^	3.0	n/d	3.0	n/d

[a] Each value represents the average of at least two duplicate titration experiments. Errors in measurements were ±0.1 with the statistical error associated with the fit of the data <0.05. n/d=not determined.

1Anion affinity constants for [**Eu.3**]^+^ were determined by plotting the change in the ratio of the Δ*J*=2/Δ*J*=0 (605–630/575–582 nm) emission bands as a function of anion concentration.


The selectivity of [**Eu.1**]^+^ and [**Eu.3**]^+^ for ATP and ADP over monophosphate anions can be attributed to increased Coulombic attraction as well as stabilizing hydrogen bonding interactions between the coordinated phosphate fragment of ATP (and ADP) and the amide N‐H groups of the quinoline chromophores (Figure 1). In support of this, [**Eu.2**]^**+**^ and [**Eu.4**]^+^, which lack amide groups, showed significantly lower affinities and essentially no selectivity between ATP, ADP, AMP and PPi. Hence, binding of NPP anions to [**Eu.1**]^**+**^ and [**Eu.3**]^**+**^ is not governed solely by favourable electrostatic interactions, but involves additional stabilization via hydrogen bonding that leads to enhanced selectivity towards ATP and ADP over monophosphate species.

Complexes [**Eu.1**]^**+**^ and [**Eu.3**]^**+**^ were found to bind to the guanosine and uridine phosphate anions with a similar level of selectivity to that determined for the adenosine phosphate series; the general order of affinity was found to be NTP≈NDP>NMP. The nucleoside moiety appears to contribute towards anion binding, as both [**Eu.1**]^**+**^ and [**Eu.3**]^**+**^ bind to NMP anions but show relatively weak affinity to HPO_4_^2−^ (log*K*_a_=2.1 and 2.7 respectively), and bind to nucleoside diphosphate anions 10 times more strongly than to pyrophosphate. Due to the lack of binding selectivity and small spectral responses exhibited for [**Eu.2**]^**+**^ and [**Eu.4**]^**+**^ with adenosine phosphate anions, affinity constants for other NPP anions were not determined.

Data in support of 1:1 binding between [**Eu.3**]^+^ and ATP or GTP was provided by high resolution (ESI) mass spectrometry, which gave intense signals at *m*/*z=*741.6588 and 749.6564, corresponding to the doubly charged species [**Eu.3**+ATP+5H]^2+^ and [**Eu.3**+GTP+5H]^2+^ respectively (Figure S50 and S51). Mass spectral data showing the formation of stable ternary adducts of [**Eu.1**]^+^ and ATP or ADP was reported previously.^31^ 2Major ESI mass spectral signals were also obtained for the ternary adducts of [**Eu.4**]^+^ bound to 1 molecule of ATP or GTP (Figures S53 and S54).


#### Effect of Mg^2+^ ions in solution

The majority of intracellular ATP exists in the form Mg‐ATP^2−^.^34^ Therefore, a cellular imaging probe for ATP should ideally be able to bind to the Mg‐ATP^2−^ complex. Indeed, enzymes that require ATP as a substrate, such as ATPases and kinases, utilize Mg^2+^ ions to provide additional stabilization of the ATP‐enzyme complex.^35^ The binding interaction between Mg^2+^ ions and ATP in water at physiological pH is approximately 50 times stronger than the interaction with ADP, with log*K*_a_ (Mg‐ATP)=4.2 and log*K*_a_ (Mg‐ADP)=3.6.^36^ The presence of Mg^2+^ ions in aqueous solution was expected to influence the ability of complexes [**Eu.1**]^**+**^ and [**Eu.3**]^**+**^ to discriminate between ATP and ADP. Indeed, previously reported synthetic receptors have shown limited discrimination between ATP and ADP at enzyme relevant concentrations of Mg^2+^ ions.^26^, ^37^ We showed recently that [**Eu.1**]^**+**^ can discriminate effectively between ATP and ADP in a buffered aqueous solution containing 3 mm Mg^2+^ ions: adding 1 mm ADP caused an 8‐fold increase in overall Eu^III^ emission intensity compared to a much smaller 2.5‐fold intensity increase in the presence of 1 mm ATP.^24^ Excellent discrimination between ATP and ADP was attributed to the slightly higher affinity of [**Eu.1**]^+^ for ADP (Table 1) as well as the higher competition between Mg^2+^ and ATP compared to ADP. This allowed the change in the ratio of ATP/ADP to be dynamically followed during the course of a kinase‐catalyzed phosphorylation reaction.

**Figure 4:**
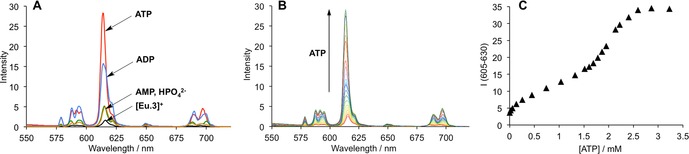
Selective spectral response of [**Eu.3**]^+^ for ATP in the presence of Mg^2+^ ions. (A) Change in emission spectra of [**Eu.3**]^+^ (5 μm) in the presence of 2 mm ATP, ADP, AMP and HPO_4_^2−^ in a fixed background of 5 mm MgCl_2_; (B) Variation in emission spectra of [**Eu.3**]^+^ upon addition of ATP (0–3 mm) in a fixed background of 5 mm MgCl_2_; (C) Increase in intensity of the Δ*J*=2 band (605–630 nm) of [**Eu.3**]^+^ as a function of ATP concentration. Conditions: 10 mm HEPES buffer, pH 7.4, *λ*_exc_=330 nm, 25 °C.

Complex [**Eu.3**]^**+**^ showed a different discriminatory behaviour between ATP and ADP in the presence of Mg^2+^ ions. Figure 4 A shows the emission spectral response of [**Eu.3**]^**+**^ in the presence of 2 mm ATP, ADP, AMP and HPO_4_^2−^ in a fixed background of 5 mm Mg^2+^ ions. Adding 2 mm ATP resulted in a substantial 24‐fold enhancement in intensity of the Δ*J*=2 emission band, whereas ADP caused a smaller 13‐fold increase in luminescence. Only minor changes in emission spectra took place in the presence of AMP and HPO_4_^2−^. Titrations of ADP and AMP into an aqueous solution containing [**Eu.3**]^+^ and 5 mm Mg^2+^ ions resulted in standard hyperbolic curves that were fitted to a 1:1 binding model (Figure S56 and S57). Apparent association constants were determined to be log*K*_a_=4.6 and 3.8 for ADP and AMP respectively, approximately 1 order of magnitude lower than those determined in the absence of MgCl_2_ (Table 2).

A titration of ATP in a fixed background of 5 mm Mg^2+^ ions generated an isotherm consistent with the occurrence of two distinct emissive species (Figure 4 C). Incremental addition of 0–1.5 mm ATP gave rise to a 4‐fold increase in emission intensity of the Δ*J*=2 band, and subsequent addition of 1.5–3.0 mm ATP induced a further 3‐fold enhancement in Eu^III^ emission, with no discernable change in spectral form. This can be ascribed to the formation of two discrete ternary adducts, each exhibiting a similar coordination environment at the Eu^III^ ion. It is hypothesized that [**Eu.3**]^+^ binds to either ATP or the Mg‐ATP^2−^ complex; with the binding geometry between [**Eu.3**]^+^ and Mg‐ATP^2−^ being similar to that of ATP, and stabilization of the negatively charged triphosphate fragment provided by the Mg^2+^ ion (Figure 1 B). During the ATP titration, the concentration of Mg‐ATP^2−^ increases, leading to the formation of a highly emissive ternary complex. High resolution mass spectrometric data supported binding of [**Eu.3**]^+^ to Mg‐ATP^2−^, giving an intense signal at 752.6439 corresponding to the doubly charged species [**Eu.3**+ATP+Mg+3H]^2+^, which was in excellent agreement with the calculated isotopic distribution (Figure S52). A titration of ATP in a fixed background of NaCl (100 mm) revealed a classic isotherm for a simple 1:1 binding system, with an apparent binding constant of log*K*_a_=5.0 (Figure S58). This confirms that the changes in emission spectra of [**Eu.3**]^+^ in the presence of Mg^2+^ ions cannot be simply a result of the additional ionic strength of the medium, rather it is the specific interactions between Mg^2+^, ATP and the Eu^III^ complex that modulates the equilibrium speciation and resulting emission response.

### Monitoring ATPase activity in real‐time

**Figure 5:**
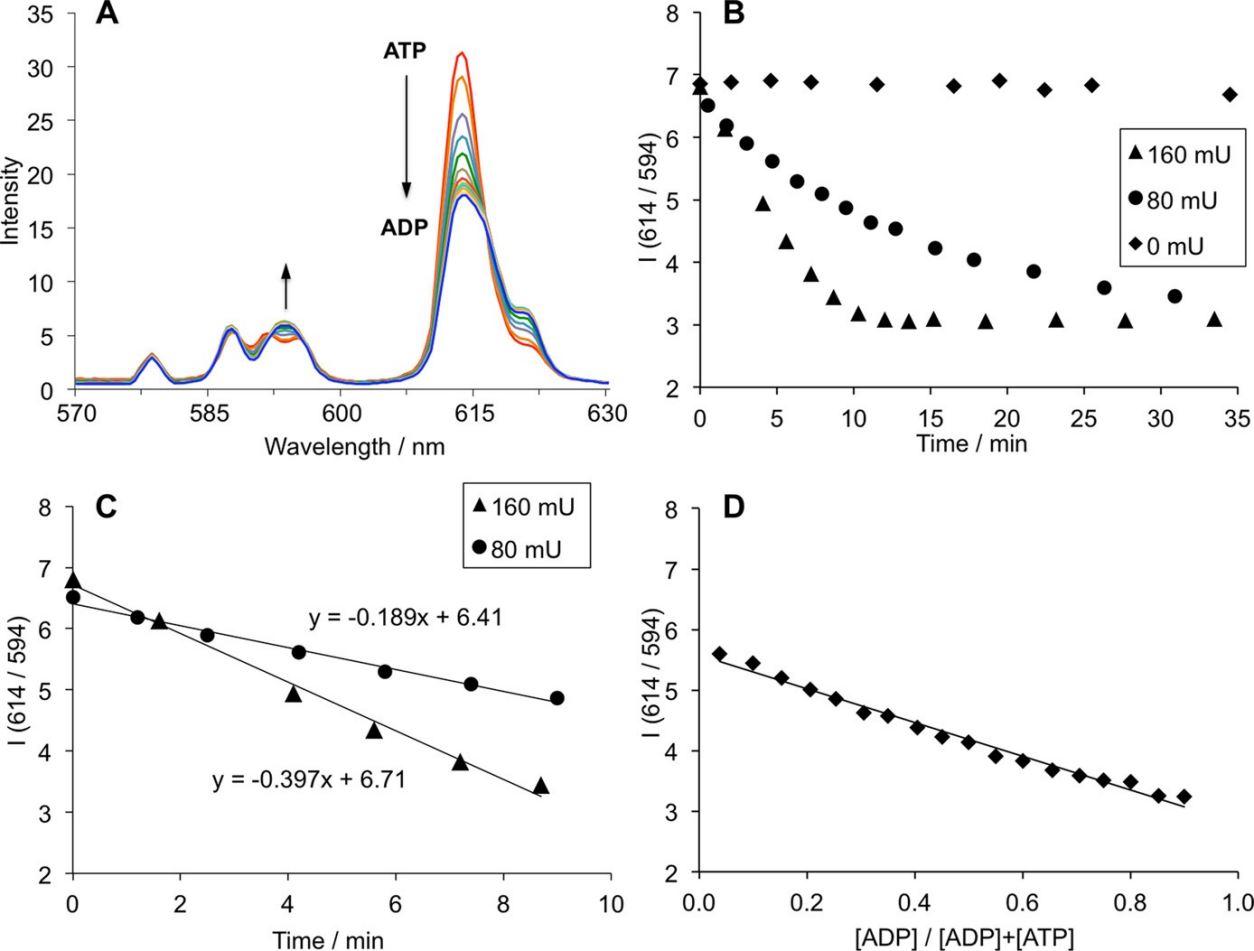
Continuous monitoring of apyrase catalyzed hydrolysis of ATP. (A) Change in emission spectra of [**Eu.3**]^+^ (570–630 nm) during the apyrase (80 mU) catalyzed hydrolysis of ATP to ADP; (B) Time‐trace plots showing the decrease in emission intensity ratio at 614/954 nm in the presence of different amounts of apyrase (0, 80 or 160 mU); (C) Linear change in emission intensity of [**Eu.3**]^+^ during the initial stages of the enzyme reaction; (D) Plot of the emission intensity ratio at 614/594 nm versus mole fraction of ADP, in the absence of apyrase (total nucleotide, [ATP]+[ADP], is constant at 2 mm). Conditions: 10 mm HEPES, pH 7.0, [**Eu.3**]^+^ (5 μm), apyrase (80 or 160 mU), ATP (2 mm), MgCl_2_ (5 mm). *λ*_exc_=330 nm, 25 °C.

Having established that [**Eu.3**]^+^ exhibits distinctive spectral responses towards ATP and ADP in a background of 5 mm Mg^2+^ ions (Figure 4 A), we demonstrated the ability of the Eu^III^ complex to monitor the apyrase‐catalyzed hydrolysis of ATP to ADP in real‐time. Apyrase is an enzyme that catalyzes the conversion of ATP into ADP, releasing energy in the process. To a buffered aqueous solution (10 mm HEPES, pH 7.0, 5 mm MgCl_2_) containing [**Eu.3**]^+^ (5 μm) and ATP (2 mm) was added different amounts of apyrase (80–160 mU, ATP/ADP selectivity ratio, 10:1). The conversion of ATP to ADP and HPO_4_^2−^ caused a time‐dependent decrease in emission intensity of the Δ*J*=2 band centred at 614 nm and a smaller increase in intensity at 594 nm within the Δ*J*=1 band (Figure 5 A). These changes in spectral form are consistent with competitive displacement of the bound ATP by ADP, as the concentration of ADP increases. Plots of the emission intensity ratio at 614/594 nm as a function of time revealed that the rate of ATP hydrolysis was directly proportional to the amount of enzyme added (Figure 5 B). The background reaction in the absence of enzyme showed essentially no change in emission intensity during the time frame of the experiment. The ratiometric changes in emission intensity observed during the enzyme reaction were in good agreement with those obtained in a simulated ATPase reaction, in which the ratio of ATP/ADP was varied systematically (Figure 5 D).

Given that [**Eu.3**]^+^ binds strongly to ATP, the possibility that the probe lowers the effective concentration of ATP must be considered, as this would influence the rate of reaction. However, anion binding to [**Eu.3**]^+^ is fast and reversible, and due to the highly sensitive nature of the luminescence response, the amount of [**Eu.3**]^+^ required to monitor the ATPase assay is much lower (5 μm) compared to the concentration of ATP (2 mm). This ensures that the rate of ATP hydrolysis is not perturbed by the presence of [**Eu.3**]^+^.

Figure 5 C shows the linear decrease in emission intensity ratio at 614/594 nm during the initial stages (0–9 minutes) of each enzyme reaction. Reducing the amount of enzyme from 160 mU to 80 mU resulted in a decrease in the initial rate of ATP hydrolysis by a factor of two. Thus, [**Eu.3**]^+^ is able to directly and continuously monitor ATPase activity by providing an instantaneous and ratiometric luminescent signal, without the need to chemically modify the enzyme or its substrate.^38^

#### Evaluating affinity to proteins

**Figure 6:**
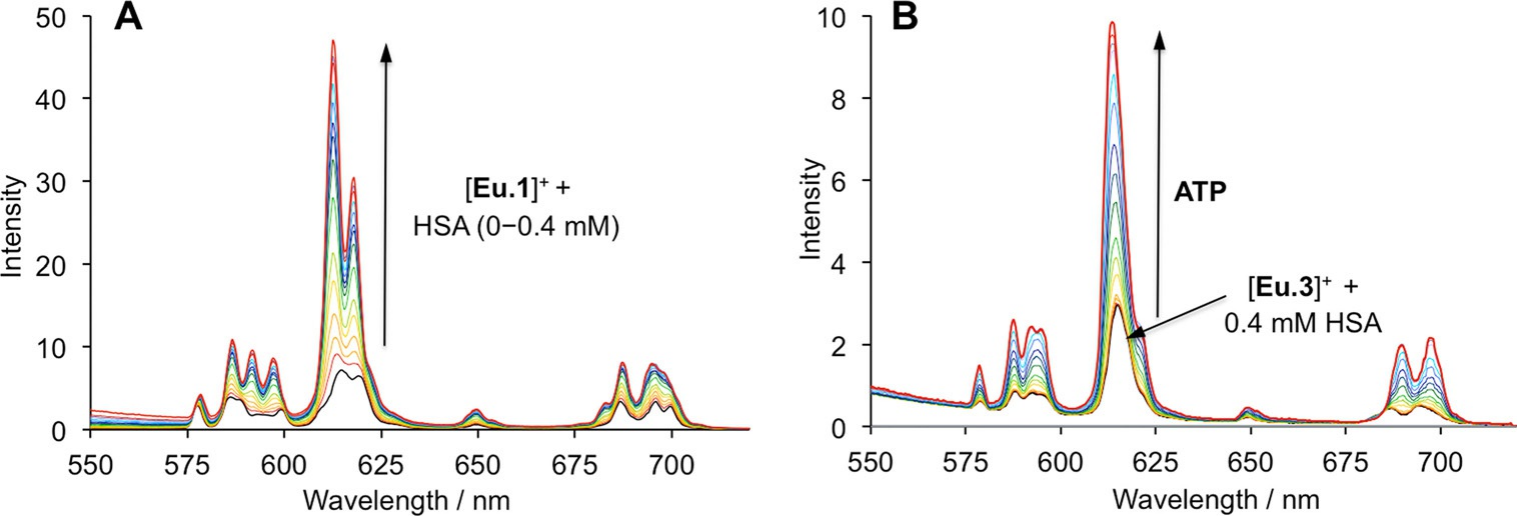
Differential responses of [**Eu.1**]^+^ and [**Eu.3**]^+^ to human serum albumin. (A) Change in emission spectra of [**Eu.1**]^+^ in the presence of increasing amounts of HSA (0‐0.4 mm); (B) Increase in emission spectra of [**Eu.3**]^+^ upon titration of ATP (0‐0.5 mm) in a fixed background of 0.4 mm HSA. Conditions: HEPES buffer (10 mm, pH 7.0), *λ*_exc_=330 nm, 25 °C.

Encouraged by the ability [**Eu.3**]^+^ to monitor the enzymatic conversion of ATP to ADP in real‐time, we wished to evaluate the ability of [**Eu.3**]^+^ to monitor fluctuations in ATP levels in living mammalian cells. A cellular imaging probe for ATP must be able to operate in the presence of proteins. Emissive metal coordination complexes are known to interact non‐covalently with proteins, often causing quenching or enhancement of emission.^39^ We measured the affinity of complexes [**Eu.1**]^+^ and [**Eu.3**]^+^ for human serum albumin (HSA), the most abundant protein in human blood plasma. Addition of HSA (0‐0.4 mm) to [**Eu.1**]^+^ at pH 7.0 resulted in a 3.5 fold increase in overall Eu^III^ emission intensity and distinctive changes in spectral form (Figure 6 A). A plot of the intensity ratio of the Δ*J*=2/ Δ*J*=1 emission bands versus HSA concentration allowed an estimation of the association constant, log*K*_a_=4.8 (*K*_d_=16 μm) (Figure S59). Luminescence lifetimes for the protein bound species in H_2_O and D_2_O were found to be 0.92 and 1.73 ms, respectively, corresponding to a hydration state, *q*=0. Thus, binding of [**Eu.1**]^+^ to HSA involves displacement of the bound water molecule, possibly by coordination of an aspartate or glutamate residue of HSA.

In sharp contrast, incremental addition of HSA to [**Eu.3**]^+^ caused a minor (10 %) increase in Eu^III^ emission intensity and no change in spectral form (Figure S60). Analysis of the luminescence lifetimes of [**Eu.3**]^+^ in the presence of HSA in H_2_O and D_2_O suggested displacement of the bound water molecule. A lower apparent binding affinity was estimated between [**Eu.3**]^+^ and HSA (log*K*_a_=3.2), which is approximately 2.5 orders of magnitude lower than the binding constant determined for this complex and ATP, under the same conditions. The minimal interaction observed between [**Eu.3**]^+^ and HSA could be tentatively attributed to the presence of two ancillary carboxylate groups in the macrocyclic ligand, which could minimize the occurrence of hydrophobic interactions between the Eu^III^ complex and the protein. Gratifyingly, addition of increasing amounts of ATP (0‐0.5 mm) to [**Eu.3**]^+^ in a fixed background of 0.4 mm HSA gave rise to a measurable 3‐fold increase in emission intensity of the Δ*J*=2 band (Figure 6 B), consistent with competitive displacement of the bound protein from [**Eu.3**]^+^, upon binding to ATP. The apparent binding constant was estimated to be log*K*_a_=4.0 (Figure S61), around two orders of magnitude lower than that determined between [**Eu.3**]^+^ and ATP in the absence of protein. Conversely, [**Eu.1**]^+^ was unable to signal the presence of ATP under the same conditions.

#### ATP recognition in simulated intracellular fluid

The above competition experiments, involving biologically relevant amounts of protein and Mg^2+^ ions, encouraged us to examine the ability of [**Eu.3**]^+^ to detect ATP in an aqueous medium that mimics the complex ionic environment within cells. In a healthy cell, the most abundant NPP anion is ATP, which is estimated to be present in concentrations between 1–5 mm (average 2.5 mm).^1^, ^12^ The majority of ATP is generated by the mitochondria by oxidative phosphorylation. Importantly, ADP is maintained at a significantly lower concentration (50–200 μm), the majority of which is bound strongly to protein, such that the ATP/ADP ratio ranges between 5 and 100.^40^ This ATP/ADP ratio acts as a critical modulator of a variety of cellular events. Other NPP anions including GTP, UTP and CTP are estimated to be present in concentrations 5‐fold lower than that of ATP. An intracellular probe must be able to respond selectively to ATP under these conditions.

**Figure 7:**
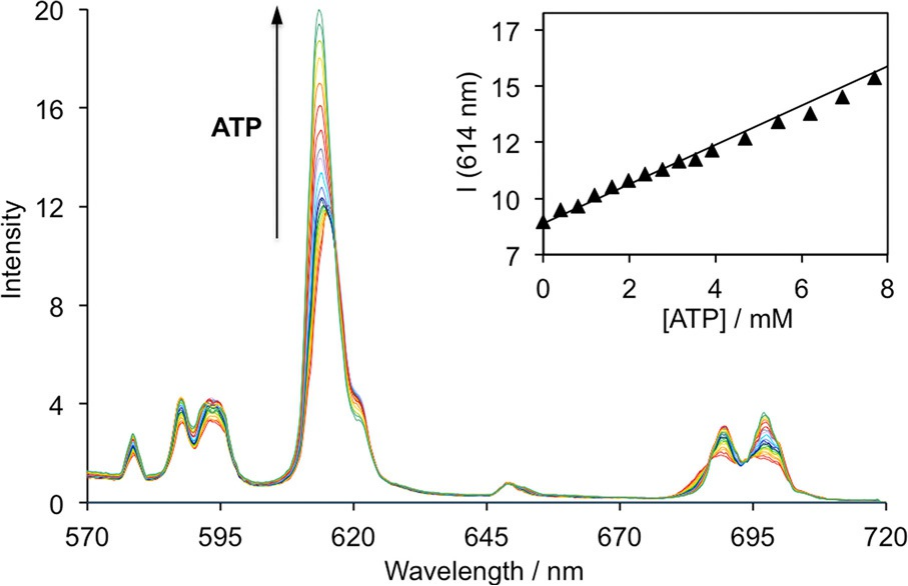
Detection of ATP in a simulated intracellular fluid. Change in emission spectra of [**Eu.3**]^+^ (50 μm) upon titration of ATP over the physiological concentration range (0–8 mm); (Inset) Linear increase in Eu^III^ emission intensity at 614 nm as a function of added ATP. Conditions: HEPES buffer (10 mm, pH 7.4), ADP (0.1 mm), GTP (0.5 mm), UTP (0.5 mm), Na_4_P_2_O_7_ (0.05 mm), Na_2_HPO_4_ (0.9 mm), NaCl (10 mm), KCl (110 mm), CaCl_2_ (2.5 mm), MgCl_2_ (8 mm), Na_2_SO_4_ (0.5 mm), sodium lactate (2.3 mm), sodium citrate (0.13 mm), glutathione (3 mm), HSA (0.4 mm). *λ*_exc_=330 nm, 25 °C.

Using the above concentrations of NPP anions, we prepared a simulated intracellular fluid based on a modified Krebs saline solution, which is commonly used in cell and tissue culture experiments. The solution contains ADP (0.1 mm), GTP (0.5 mm) and UTP (0.5 mm), NaH_2_PO_4_ (0.9 mm), PPi (0.05 mm), KCl (110 mm), NaCl (10 mm), CaCl_2_ (2.5 mm), MgCl_2_ (8 mm), Na_2_SO_4_ (0.5 mm), sodium lactate (2.3 mm), sodium citrate (0.13 mm), glutathione (3 mm) and HSA (0.4 mm), buffered at pH 7.4 using 10 mm HEPES. Under these conditions, incremental addition of 0–6 mm ATP to [**Eu.3**]^+^ (50 μm) resulted in a reproducible 55 % enhancement in emission intensity of the Δ*J*=2 band centred at 614 nm (Figure 7). Crucially, the increase in Eu^III^ emission intensity was approximately linear over a biologically relevant ATP concentration range of 0.3 to 8.0 mm, corresponding to an increase in the ATP/ADP ratio from 3 to 80 (Figure 7, inset). These competition experiments strongly indicated that [**Eu.3**]^+^ could be used to signal changes in the ATP/ADP ratio in cellulo, allowing a range of biological processes to be followed in real‐time.

Before undertaking cellular imaging studies, the pH dependence of the emission response of [**Eu.3**]^+^ in the presence of 2 mm ATP was assessed. The emission intensity and spectral form was essentially unchanged between pH 6.4 and 8.6 (Figure S62), suggesting that the luminescence signal should not be affected by pH fluctuations around normal mitochondrial or cytoplasmic pH, estimated to be near 7.3 and 8.0, respectively.^34^ This is significant, as previously reported ATP‐selective imaging probes have been shown to be sensitive to changes in intracellular pH.^6^, ^7^ Additionally, certain existing methods for measuring intracellular ATP are dependent on dissolved oxygen concentration (e.g. the bioluminescent luciferase reaction).^9^ To confirm that [**Eu.3**]^**+**^ is not sensitive to changes in dissolved oxygen, emission spectra were recorded for [**Eu.3**]^**+**^ alone, and in the presence of 2 mm ATP, in air‐equilibrated and degassed aqueous solution. The Eu^III^ complex showed less than 5 % variation in emission intensity under these conditions (Figure S63), with no significant change in emission intensity being observed after bubbling of the air‐equilibrated sample with oxygen gas for 10 minutes.

### Imaging mitochondrial ATP in living cells

#### Cellular uptake and localization studies

**Figure 8:**
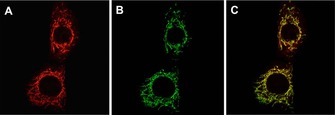
LSCM images showing mitochondrial localization of the Eu^III^ complex. (A) Localization of [**Eu.3**]^+^ (50 μm) in the mitochondria of NIH‐3T3 cells (*λ*_exc_ 355 nm, *λ*_em_ 605–720 nm); (B) MitoTracker Green (*λ*_exc_ 488 nm, *λ*_em_ 500–530 nm); (C) RGB merged image verifying co‐localization (*P*=0.91).

The cellular uptake behaviour of [**Eu.3**]^+^ was examined in NIH‐3T3 cells using fluorescence and laser scanning confocal microscopy (LSCM).^42^ Incubation of [**Eu.3**]^+^ (50 μm) in NIH‐3T3 cells for 2 hours resulted in uptake of the Eu^III^ complex and predominant localization to the mitochondria (*λ*_exc_ 355 nm, *λ*_em_ 605–720 nm), verified by co‐localization studies using MitoTracker Green (*λ*_exc_ 488 nm, *λ*_em_ 500–530 nm, Pearson's correlation coefficient, P=0.91) (Figure 8). The preferential distribution of [**Eu.3**]^+^ in the mitochondria could be tentatively attributed to the overall positive charge of the Eu^III^ complex and the amphipathic nature of the macrocyclic ligand.^16^ Imaging was possible over extended time periods (up to 8 hours), during which time the brightness of the observed images did not vary significantly (±10 %) and the cells appeared to be healthy and proliferating (see supporting information for detailed analysis of probe brightness within cells).

Cytotoxicity and vitality studies were undertaken for [**Eu.3**]^+^ at 24 hours using image cytometry assays, involving DAPI and Acridine Orange stains, which revealed an IC_50_ value of greater than 200 μm. Considering that the incubation concentration of [**Eu.3**]^+^ is four times lower than this value, it can be assumed that the Eu^III^ complex is essentially non‐toxic during the time frame of the imaging experiments. Analysis of ICP‐MS data showed that for 4×10^6^ NIH‐3T3 cells incubated with [**Eu.3**]^+^ (50 μm) for 2 hours, a given cell contained 69 μm (±5 %) of Eu^III^ metal, consistent with the accumulation of [**Eu.3**]^+^ within the mitochondria during the incubation period.

#### Imaging elevated mitochondrial ATP levels

**Figure 9:**
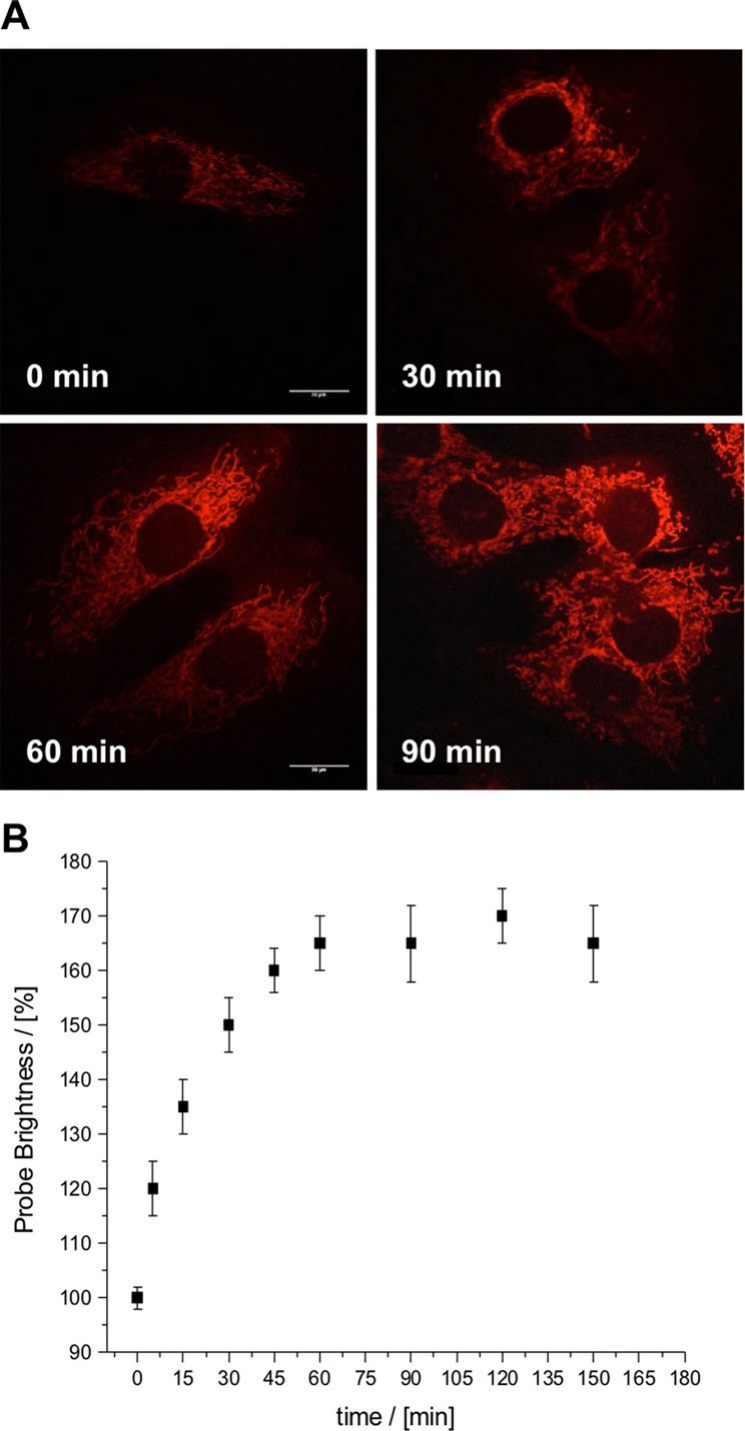
Real‐time monitoring of elevated levels of ATP in mitochondria. (A) Time‐lapsed LSCM images of NIH‐3T3 cells stained with [**Eu.3**]^+^ (50 μm, *λ*_exc_ 355 nm, *λ*_em_ 605–720 nm) before (0 min) and after treatment with staurosporine (10 nm), a potent inhibitor of protein kinases; (B) Time‐dependent increase in emission intensity (605–720 nm) of cells stained with [**Eu.3**]^+^ following treatment with staurosporine (10 nm).

We evaluated the ability of [**Eu.3**]^+^ to visualize changes in the concentration of ATP in NIH‐3T3 cells upon treatment with staurosporine, a broad‐spectrum inhibitor of kinase activity that eventually induces cell apoptosis. Figure 9 A shows time‐lapsed images of cells stained with [**Eu.3**]^+^ (50 μm) before (0 min) and after treatment with staurosporine (10 nm). The images revealed a gradual increase in the observed emission intensity (*λ*_em_ 605–720 nm) in the mitochondria over a 90 minute period. At 60 minutes post‐staurosporine treatment, the Eu^III^ emission intensity had increased by approximately 65 %, after which time the observed signal reached a plateau (Figure 9 B). No obvious signs of cell apoptosis were evident during the time scale of the experiment, and the Eu^III^ complex remained localized to the mitochondria. As a control, cells incubated with [**Eu.3**]^+^ without the addition of staurosporine were examined, revealing less than 10 % variation in the Eu^III^ emission intensity over the same time period (Figure S64). ICP‐MS studies of cells incubated with [**Eu.3**]^+^ and staurosporine for 2 hours revealed essentially no change in the concentration of accumulated Eu^III^ metal, compared to cells grown in the absence of the kinase inhibitor (Figure S65). Therefore, treatment with staurosporine does not appear to perturb cellular uptake or efflux of the Eu^III^ complex from cells.

These imaging experiments indicate that the concentration of ATP in the mitochondrial region gradually increased during the preapoptotic period (150 min) following treatment with staurosporine. This is in agreement with previous reports of elevated ATP levels in the cytosol^7^ and mitochondria5a upon incubation with staurosporine. The mitochondria remained intact during this time, suggesting that mitochondrial functional integrity is important during the early stages of apoptosis. This is consistent with the notion that apoptosis is an energy requiring process; the concentration of mitochondrial ATP increases to supply chemical energy for a variety of intracellular processes, such as enzymatic hydrolysis of macromolecules.^7^

#### Monitoring depleted ATP levels in living cells

**Figure 10:**
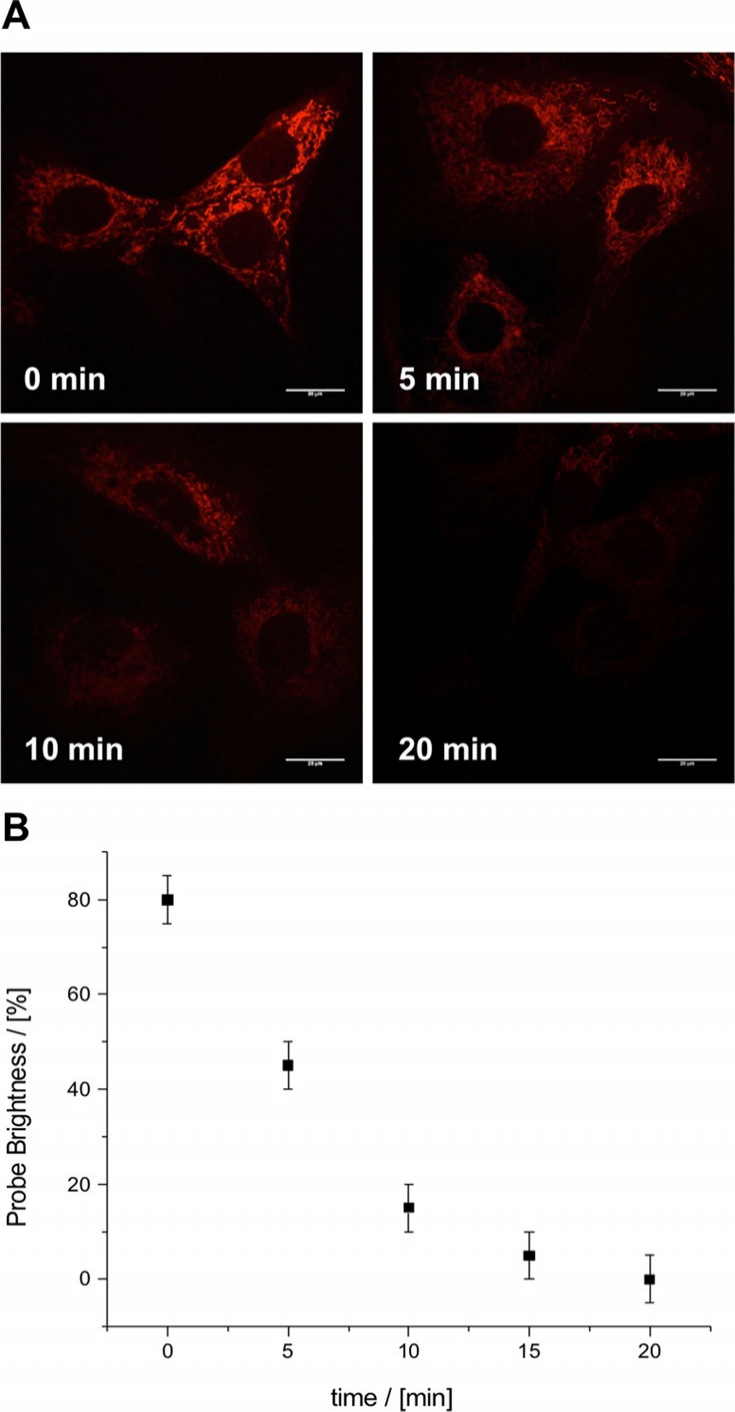
Real‐time monitoring of depleted mitochondrial ATP levels. (A) Time‐lapsed LSCM images of NIH‐3T3 cells stained with [**Eu.3**]^+^ (50 μm, *λ*_exc_ 355 nm, *λ*_em_ 605–720 nm) under glucose starvation conditions, before (0 min) and after treatment with KCN (0.1 mm), an inhibitor of oxidative phosphorylation; (B) Time dependent decrease in emission intensity of cells stained with [**Eu.3**]^+^ following treatment with KCN (0.1 mm).

Next, the change in mitochondrial ATP levels was monitored after treatment of cells with potassium cyanide, an inhibitor of oxidative phosphorylation.6a Initially, NIH‐3T3 cells were incubated with [**Eu.3**]^+^ in a glucose‐free growth medium, in order to inhibit ATP production via glycolysis. Under these conditions, a 20 % decrease in emission intensity was observed after 2 hours compared to cells grown in the presence of glucose (Figure S66). Subsequent addition of potassium cyanide (0.1 mm) resulted in a significant and rapid decrease in the observed emission intensity, with almost 85 % of luminescence lost after 10 minutes (Figure 10). These results suggest that mitochondrial ATP is depleted substantially in the presence of KCN, due to inhibition of oxidative phosphorylation, which is consistent with previous studies that show a reduction in intracellular ATP under similar conditions.6a ICP‐MS analysis of cells incubated with [**Eu.3**]^+^ for 30 minutes with KCN under glucose starvation conditions showed less than 10 % variation in the concentration of accumulated Eu^III^ metal, relative to untreated cells grown in the presence of glucose. Therefore, the possibility that incubation with KCN promotes efflux of [**Eu.3**]^+^ from the cells can be ruled out.

The inhibition of oxidative phosphorylation may result in perturbation of mitochondrial bicarbonate concentration, as bicarbonate stimulates the enzymatic production of cyclic AMP, which in turn activates mitochondrial kinase activity, regulating ATP production.^43^ Having already shown that the binding of bicarbonate to [**Eu.3**]^+^ is approximately 100 times weaker than that of ATP in buffered aqueous solution (Table 2), we wished to investigate further the effect of varying intracellular bicarbonate concentration on the emission intensity of [**Eu.3**]^+^. Following a procedure described previously,19a NIH‐3T3 cells were loaded with [**Eu.3**]^+^ (50 μm) and the percentage of atmospheric CO_2_ in the cell imaging chamber was varied between 2 % and 7 % CO_2_. The Eu^III^ emission intensity observed in the mitochondria of NIH‐3T3 cells after an incubation period of 60 minutes was compared to those obtained when the incubation was performed at a constant atmospheric CO_2_ of 5 % for the same time period. The LCMS images revealed less than 5 % modulation of emission intensity upon variation of the atmospheric CO_2_ (Figure S67). These microscopy experiments indicate that [**Eu.3**]^**+**^ does not respond significantly to fluctuations in the equilibrium cellular bicarbonate concentration, which is expected considering the lower apparent binding constant of [**Eu.3**]^**+**^ to bicarbonate, and the larger emission intensity of the ATP‐bound Eu^III^ complex.

Taken together, these live‐cell imaging experiments demonstrate that [**Eu.3**]^+^ is capable of signalling changes in the concentration of ATP in the mitochondria upon incubation with an ATP synthesis inhibiter (KCN) and a kinase activity inhibitor (staurosporine), and could provide a versatile imaging tool for studying cell metabolism and other ATP‐requiring processes in real‐time, within a targeted organelle.

## Conclusions

We have developed a discrete, cationic Eu^III^ complex for monitoring dynamic changes in the concentration of ATP within the mitochondria of living cells. A series of luminescent Eu^III^ complexes, [**Eu.1**–**4**]^+^, was synthesised that bind reversibly to nucleoside polyphosphate anions with differential affinities in buffered aqueous solution at physiological pH. The affinity of the Eu^III^ complexes towards NPPs and the magnitude of the emission spectral response is tunable by making modifications to the ligand structure. Hydrogen bond donor groups were introduced into the quinoline units of [**Eu.1**]^+^ and [**Eu.3**]^+^ to enhance selectivity towards ATP and ADP over monophosphate anions. Complex [**Eu.3**]^+^, bearing two neutral carbonyl amide donors, binds most strongly to ATP (log*K*_a_=5.8), forming a stable ternary complex that exhibits intense, long‐lived Eu^III^ luminescence. [**Eu.3**]^+^ can discriminate effectively between ATP, ADP and AMP in a competitive aqueous medium that simulates the complex ionic environment present in cells. The probe provides a linear, ratiometric emission response that is proportional to the ratio of ATP/ADP, enabling the enzymatic hydrolysis of ATP to ADP to be precisely monitored in real‐time.

Cellular localization studies revealed that [**Eu.3**]^+^ preferentially stains the mitochondria of mammalian cells, and is retained within this organelle over extended time periods. We have shown that [**Eu.3**]^+^ can detect an increase in mitochondrial ATP concentration following treatment of cells with the kinase inhibitor staurosporine. Additionally, [**Eu.3**]^+^ was able to visualize a rapid decrease in mitochondrial ATP following treatment with KCN, an inhibitor of oxidative phosphorylation. Complex [**Eu.3**]^+^ offers a several advances in performance compared to existing ATP‐responsive probes, including a luminescence signal that is: 1) sensitive to ATP within the biologically relevant concentration range (1–5 mm); 2) minimally perturbed by changes in pH, dissolved oxygen or the presence of protein; and 3) sufficiently long‐lived to avoid interference from UV‐induced autofluorescence arising from biomolecules. Such probes offer a new versatile tool for studying metabolism and a range of biological processes involving ATP, with subcellular resolution. The strategy employed here will be explored further to design Eu^III^ probes capable of monitoring spatio‐temporal ATP dynamics within different cellular compartments, providing a ratiometric change in emission intensity that is intrinsically normalized.

## Experimental Section

Comprehensive experimental details are provided in the supporting information, including: details of compound characterization, UV/Vis and emission spectral data for [**Eu.1**–**4**]^+^, luminescence titration experiments involving [**Eu.1**–**4**]^+^ and NPP anions (conducted in buffered aqueous solution and competitive aqueous media), pH dependence of the emission profile of [**Eu.3**]^+^, ICP‐MS data, cellular imaging analysis and selected LSCM images of [**Eu.3**]^+^ in NIH‐3T3 cells.

## Conflict of interest

The authors declare no conflict of interest.

## Supporting information

As a service to our authors and readers, this journal provides supporting information supplied by the authors. Such materials are peer reviewed and may be re‐organized for online delivery, but are not copy‐edited or typeset. Technical support issues arising from supporting information (other than missing files) should be addressed to the authors.

Supplementary
