# Supplementary material for: Cationic Europium Complexes for Visualizing Fluctuations in Mitochondrial ATP Levels in Living Cells
Source: Chemistry. 2018 Jun 28;24(42):10745–55. doi: 10.1002/chem.201801008 (PMC6175470; doi:10.1002/chem.201801008)
Supplement: Supplementary file 1 — Supplementary [file CHEM-24-10745-s001.pdf]

Author Contributions

R.M. Investigation: Lead
T.T. Investigation: Supporting.
